# Automation and Optimization of Food Process Using CNN and Six-Axis Robotic Arm

**DOI:** 10.3390/foods13233826

**Published:** 2024-11-27

**Authors:** Youngjin Kim, Sangoh Kim

**Affiliations:** Department of Food Engineering, Dankook University, 119, Dandae-ro, Dongnam-gu, Cheonan-si 31116, Chungcheongnam-do, Republic of Korea; jiny@dankook.ac.kr

**Keywords:** robotic arm, food processing, coffee roasting, automation, optimization, computer vision, convolutional neural network

## Abstract

The Food Process Robot Intelligent System (FPRIS) integrates a 3D-printed six-axis robotic arm with Artificial Intelligence (AI) and Computer Vision (CV) to optimize and automate the coffee roasting process. As an application of FPRIS coffee roasting, this system uses a Convolutional Neural Network (CNN) to classify coffee beans inside the roaster and control the roaster in real time, avoiding obstacles and empty spaces. This study demonstrates FPRIS’s capability to precisely control the Degree of Roasting (DoR) by combining gas and image sensor data to assess coffee bean quality. A comparative analysis between the Preliminary Coffee Sample (PCS) and Validation Coffee Sample (VCS) revealed that increasing roast intensity resulted in consistent trends for both samples, including an increase in weight loss and Gas sensor Initial Difference (GID) and a decrease in Sum of Pixel Grayscale Values (SPGVs). This study underscores the potential of FPRIS to enhance precision and efficiency in coffee roasting. Future studies will expand on these findings by testing FPRIS across various food processes, potentially establishing a universal automation system for the food industry.

## 1. Introduction

Colorimeters and spectrophotometers are widely used in food color analysis, yet Artificial Intelligence (AI)-based computer vision (CV) technology is increasingly being applied to monitor color changes during food processing [1]. Machine Learning (ML), a broad category of statistical techniques within AI, enables computer programs to learn to associate data with predictive capabilities [2]. Deep Learning (DL), a subset of ML, utilizes deep neural networks to build complex architectures and generate models through repeated function application across multiple layers [3]. Convolutional Neural Networks (CNNs), a type of DL, have demonstrated significant potential in image classification tasks, especially in food industry applications [4]. Previous studies have investigated various applications of AI in the food industry: They have [5] developed a model to estimate the shelf life of moisture-sensitive foods; [6] integrated sensor systems with ML to evaluate beer quality in real time; [7] applied image augmentation for tomato pest identification; [8] used ML with image spectroscopy to assess potato quality; and [9] optimized freezer energy consumption using ML. AI and ML play crucial roles in food quality assessment and process automation. These technologies are also applicable to the coffee roasting process, utilizing CNNs to quantify roasting based on color changes. This approach enables the optimization of the roasting process, ultimately reducing labor costs and increasing productivity [10].

Modern food processing and manufacturing plants face the challenge of meeting the growing food demand of an increasing population by producing more at lower costs in a sustainable manner [11]. This demand has heightened the need for efficient automation systems in the food industry. Robotics is a key component of agricultural and industrial automation [12]. In food processing, robots are primarily used for pick-and-place operations, performing tasks such as sorting, packing, and packaging [13]. Robotic automation is most effective in addressing specific manufacturing and processing challenges [14]. In the food and beverage manufacturing industry, robotic automation provides numerous benefits, with flexibility as its most vital advantage. Robots enhance product quality and consistency by performing planned tasks with precision and repetition. Moreover, the risk of workplace injuries from repetitive motions is reduced, improving overall working conditions. Increased efficiency lowers production costs and time while minimizing waste [15].

Coffee is native to the African continent and the island of Madagascar, with approximately 80 known species [16]. The two primary commercial coffee species are *Coffea arabica* (Arabica coffee) and *Coffea canephora* (including Conilon and Robusta varieties) [17]. These coffees are prepared into beverages through the roasting process, which involves heating green coffee beans for 12–20 min at temperatures between 200 °C and 300 °C [18]. Roasting is the most crucial step in transforming green coffee beans from tasteless and aromatic into flavorful coffee. The quality and chemical composition of coffee beans primarily depend on the Degree of Roasting (DoR), controlled by roasting time and temperature. In coffee production, distinguishing poorly roasted beans due to uneven conditions from those with optimal DoR is essential to avoid undesirable quality [19]. Generally, DoR assessment is conducted globally through organoleptic evaluation by coffee experts, bean reflectivity measurements, or color analysis [20].

Previous roasting studies include [21], which investigated changes in caffeine, trigonelline, chlorogenic acid, and monosaccharide content during roasting, and [22], which examined differences in solids and polyphenol content between medium and strong roasts. Another study, [23], analyzed variations in pH, acidity, solids, and caffeine content by roasting stage, while [24] measured antioxidant activity based on roasting temperature.

The food tech market, which adds value by applying Internet of Things (IoT), AI, robotics, and 3D printing to the food industry, is expanding [25]. However, research on developing food tech technologies applicable to the coffee roasting process remains limited. In this study, the Food Process Robot Intelligent System (FPRIS) integrates a 3D-printed 6-axis robotic arm with sensor data (gas and image) using computer vision technology with a CNN. FPRIS is designed to classify coffee bean images while avoiding empty spaces and obstacles inside the rotating roaster. By integrating classified images with gas sensor data, FPRIS controls food processing machinery to quantify coffee bean quality in real time, ultimately optimizing and automating the roasting process through the robotic arm. This paper is structured as follows: Section 2 describes the materials and methods used in the development and implementation of the FPRIS. Section 3 presents the results obtained from the experiments and provides a detailed discussion on their implications. Finally, Section 4 concludes the study by summarizing the key findings and offering perspectives for future research.

### Literature Review

The application of AI in the food industry has been growing over the years for a variety of reasons, such as food classification, parameter classification and prediction, quality control, and food safety. Of these, in the food processing industry, AI can be used to optimize food processing operations such as sorting and grading, and to detect defects and contaminants in food. This can improve the quality and consistency of food and reduce waste. AI can also be used to analyze individual consumer data, such as age, gender, and activity level, to provide personalized nutrition recommendations. This can help consumers make more informed choices about their diet and improve their overall health [26].

Ref. [2] developed an adaptive bread-making machine using ML to optimize the baking process. The team designed a Baking Process Prediction Model (BPPM) that monitors and analyzes real-time sensor and vision data to accurately predict baking stages and improve bread quality. Bread baked using BPPM exhibited larger volume and better quality compared to bread baked with fixed times, highlighting the potential of AI-based systems to enhance precision and efficiency in the food processing industry.

Ref. [27] explored the potential application of AI in detecting mycotoxin contamination in foods. Mycotoxins, toxic substances produced by fungi, pose significant threats to food safety and public health. Traditional detection methods are time-consuming and costly, limiting their practicality. AI techniques, utilizing data from spectroscopy and hyperspectral imaging, demonstrated the potential to enhance detection efficiency and improve food safety.

Ref. [28] proposed a method combining Hyperspectral Imaging (HSI) and DL for the rapid and non-destructive quality assessment of chestnuts. HSI data within the 935–1720 nm range were analyzed, and the FD-LSTM model achieved a high accuracy of 99.72%. The study identified specific wavelengths (1000, 1400, and 1600 nm) as critical for quality evaluation, highlighting the effectiveness of integrating HSI and DL as an efficient quality detection tool.

Ref. [29] studied the combination of HSI and DL to detect adulteration in red meat. They utilized data obtained considering different meat states (fresh, frozen, thawed, and packaged) and achieved a high accuracy of 94.4% using a CNN model. This study shows that the combination of HSI and DL has the potential to be a reliable non-destructive analysis tool for meat adulteration detection.

Ref. [30] explored the use of hyperspectral imaging combined with a CNN to identify different rice seed varieties. They acquired hyperspectral images of four rice seed varieties across two spectral ranges: 441–948 nm and 975–1646 nm. The study compared the performance of K-Nearest Neighbors (KNNs), Support Vector Machines (SVMs), and CNN models, finding that CNNs generally outperformed the other methods. The results suggest that CNNs are effective for analyzing spectral data in rice seed variety identification.

Ref. [31] applied machine learning and image processing techniques for the quantitative analysis of industrial fermentation processes. A CNN-based model was developed to analyze foam images from the fermentation process, enabling the real-time measurement of fermentation progress and demonstrating the potential of AI-based technologies in the fermentation food industry.

These studies highlight how versatile and effective AI is for solving a variety of challenges in food processing, from quality grading to defect detection.

In food processing, robots are primarily used for pick-and-place operations to complete tasks such as sorting, wrapping, and packaging. Robotic automation is most effective when implemented to solve or improve specific manufacturing and processing scenarios. It offers many benefits to food and beverage manufacturing, the most important and significant of which is flexibility. By its very nature, robotics offers reconfigurability and quick adaptation to new work environments and new processes [32].

Ref. [33] proposed a robotic end-effector evaluation system for food handling. The investigators conducted pick-and-place tests using commercial and under-development robotic end-effectors and proposed a scoring system for evaluating the performance of robotic end-effectors.

Ref. [34] introduced the challenges of robotic food handling in the food industry. The latest advances in robotic end-effectors, food recognition, and basic information about food were reviewed. A key challenge is the development of practical, low-cost robotic end-effectors that can scope with a wide range of food products.

Ref. [35] is a comprehensive review of the prospects for robotics in the food processing industry based on 50 papers. Key findings include the integration of industrial robots throughout the food manufacturing sector, increasing production rates, improving food quality and hygiene, and highlighting the future prospects for food robots and the need to develop optimized protocols.

Ref. [33] proposed a performance evaluation system for robotic end-effectors capable of handling various food products. The study categorized foods based on their physical properties and classified end-effectors by their gripping methods, evaluating seven commercial and under-development end-effectors using 14 types of real food items and their samples. This approach enabled a quantitative comparison of the performance of each end-effector and visually demonstrated their applicability in processing diverse food products.

Ref. [36] evaluated the implementation of robotic automation systems in the loading process of Home Meal Replacement (HMR) products to improve production efficiency. Two scenarios—mass production and order-based production—were simulated to analyze bottlenecks, worker utilization, and throughput. The results showed that robotic automation increased throughput by 31.2% in mass production and 12.0% in order-based production. This study provides critical insights into enhancing productivity, reducing labor dependency, and fostering digital innovation in food manufacturing through robotics integration.

Ref. [37] proposed a robotic system for clearing tableware in self-service restaurants. The robot utilizes various sensors to detect tableware and leftover food on tables, efficiently collecting and transferring them to dishwashers. This system has the potential to reduce operation time and enhance the efficiency of self-service restaurant operations.

Ref. [38] developed a portable Meal-Assistance Robot (MAR) designed to help patients eat independently, reducing the need for caregiver involvement. The system features a 7-degree-of-freedom robotic arm, a food tray, and a tablet-based interface tailored for Korean-style meals. Using DL-based instance segmentation, it identifies food acquisition points and incorporates a face recognition interface for users with severe disabilities. This innovation aims to ease caregiver workloads and enhance the autonomy and quality of life for individuals requiring meal assistance.

These studies show that advanced technologies and AI play an important role in solving various problems in the food industry. In particular, AI-based analytical techniques have demonstrated their potential to be used as a variety of non-destructive tools, while robotic automation contributes to increasing work efficiency and flexibility. Building on these technological advances, this research aims to integrate robotics and AI into one of the food processing processes, coffee roasting, to achieve optimization and automation in the process.

## 2. Materials and Methods

### 2.1. Six-Axis Robotic Arm Design

The six-axis robotic arm developed in this study utilizes a total of six brushless motors (CubeMars, Jiangxi, China), each designed to provide the degrees of freedom necessary for the arm to move at various angles. Additionally, the gripper is equipped with a stepper motor (28BYJ-48, Changzhou Fulling Motor Co., Ltd., Changzhou, China), which enables precise gripping movements. A camera (ELP-USBFHD08S-MFV, Shenzhen Ailipu Technology Co., Ltd., Shenzhen, China) with a resolution of 640 × 360 pixels and a frame rate of 260 frames per second is mounted on the robotic arm to capture visual data, which are then analyzed for control purposes. The detailed specifications of the motors used in both the robotic arm and the gripper are shown in Table 1.

The main structural components of the robotic arm were fabricated using the Creality K1 3D printer (Creality, Shenzhen, China) and Hyper PLA_1.75 filament (Creality, Shenzhen, China). This approach enabled the lightweight construction and precise implementation of the robotic arm’s design. The structural design of the robotic arm was created using the Computer-Aided Design (CAD) application Rhino 8 (McNeel, Seattle, WA, USA). After designing each part, the models were exported as Standard Tessellation Language (STL) files. The slicer software, Creality Print (Version 4.3.8), was then used to convert these STL files into g-code files. This process prepared the models for 3D printing, ensuring precise instructions for the printer to fabricate the components accurately.

**Figure 1:**
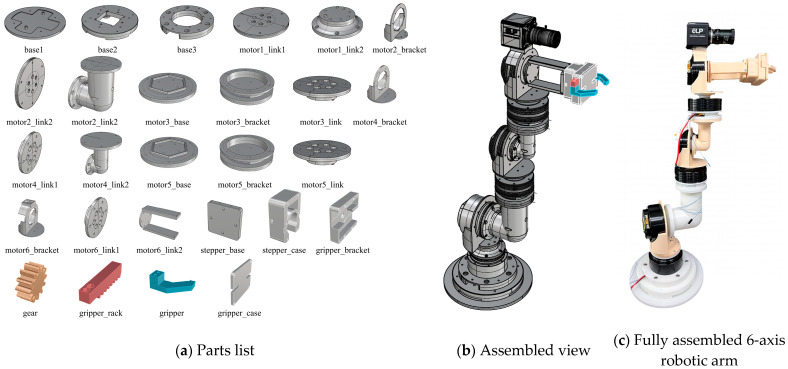
The 6-axis robotic arm designed.

The robotic arm designed in this study is shown in Figure 1. All components were printed using the same parameters: The resolution was set to 0.2 mm, with an infill density of 60%. A zig-zag pattern was used for support structures, and the nozzle and bed temperatures were maintained at 220 °C and 45 °C, respectively, for all parts. Additionally, the slicing data from Creality Print, as shown in the G-code analysis, are summarized in Table 2, detailing the printing time, material weight, and material length for the robotic arm components.

The control of the six-axis robotic arm was implemented using an Arduino (Uno, Arduino Co., New York, NY, USA) and a Controller Area Network (CAN) communication module (MCP2515, Microchip Technology Inc., Chandler, AZ, USA). The CAN bus is a serial bus with a maximum bit rate of 1 Mbit/s, known for its high reliability, real-time transmission, adaptability, and cost-effectiveness. It is widely used in various fields such as industrial automation, automotive, and robotics [39].

### 2.2. Green Coffee Bean and Roaster

In this study, green coffee beans (Essentia Factory Co., Gimhae-si, Republic of Korea), an heirloom variety of *Arabica* grown at an altitude of 2150 m in the Yirgacheffe region of Ethiopia and naturally processed, were purchased and utilized. The green coffee beans were vacuum-packed to prevent spoilage and stored in a refrigerator (CMF-TK40, Foschan Great Power Co., Ltd., Goyang-si, Republic of Korea) at 21 °C with 40% relative humidity [40]. The weight of the coffee beans was measured using a precision scale (GB303, Mettler Toledo Inc., Greifensee, Switzerland). Roasting was performed using a roaster (CBR-101A, Genecafe Co., Ansan-si, Republic of Korea) that employs a three-dimensional rotary twisting method and is heated by indirect hot air.

### 2.3. BeanFinder CNN (BFCNN)

#### 2.3.1. Dataset Preparation for the BFCNN

**Figure 2:**
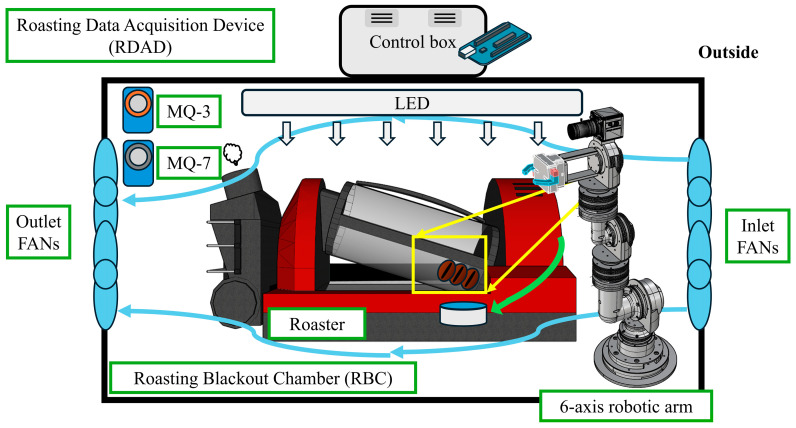
Roasting Data Acquisition Device (RDAD) with Roasting Blackout Chamber (RBC).

To collect raw data, a Roasting Blackout Chamber (RBC) was constructed to block external light interference. For consistent illumination, two LED lights (LM52045M30-LS, Lumenlux Co., Bucheon-si, Republic of Korea) were installed inside the RBC, achieving a Color Rendering Index (CRI) of over 80 Ra. To ensure efficient heat dissipation, four fans (MGA9212HR-A25, Protechnik, Geumcheon-gu, Seoul, Republic of Korea) were installed, with two fans positioned at the inlet and two at the outlet, each providing an airflow of 2 × 1.369 CMM. The structure of the RBC is shown in Figure 2. Raw data were collected by capturing images of the rotating roaster using a high-speed camera mounted on the six-axis robotic arm. The camera was connected to a workstation via USB for data transmission.

#### 2.3.2. Dataset Collection for the BFCNN

**Figure 3:**
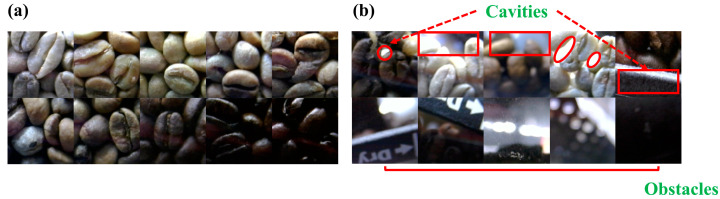
(**a**) Coffee bean images; (**b**) non-coffee bean images with cavities and obstacles.

The experiment was conducted on a laptop (Lenovo V14 G2 ALC, Lenovo Group Ltd., Beijing, China) running Windows 11 (22H2) and Python (version 3.8.10). The laptop was equipped with an 11th Gen Intel(R) Core(TM) i5-11300H CPU and an NVIDIA GeForce RTX 3050 Laptop GPU. A total of 100 g of green coffee beans were placed in the roaster and roasted at 200 °C for 60 min. During the roasting process, images with a resolution of 640 × 360 were captured every 0.2 s. The training images, sized 250 × 250 pixels, were created by cropping the region of interest from the raw images. These images were then labeled as either coffee bean or non-coffee bean. The dataset was randomly divided into training, validation, and evaluation sets in a ratio of 8:1:1 using the splitfolders class from the Python library. The images were scaled to a range of 0 to 1 by dividing by 255 using the ImageDataGenerator class from Keras (version 2.12.0) for real-time image data augmentation. To further increase the diversity of the training dataset, the images were augmented by rotating them 10° and randomly flipping them both vertically and horizontally, following the method described by [41]. A sample of the training data is shown in Figure 3.

#### 2.3.3. The Design of BFCNN

**Figure 4:**
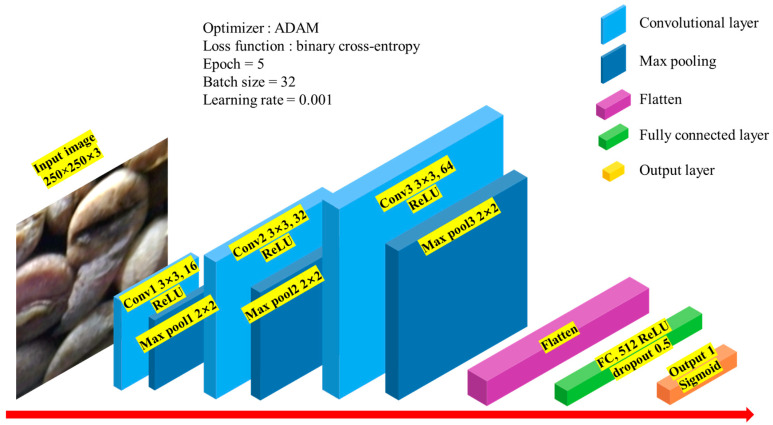
Structure of the BFCNN.

TensorFlow (version 2.12.0) and Keras were utilized as the DL frameworks for implementing the BFCNN. The architecture of the neural network is shown in Figure 4. The input images for the CNN-based BFCNN have dimensions of 250 × 250 pixels. The first convolutional layer (Conv1) uses a 3 × 3 filter to generate 16 feature maps, with the Rectified Linear Unit (ReLU) activation function applied.

The ReLU activation function is defined by the following equation [42]:

(1) $$y=\left\{\begin{array}{c} x,\;x>0\\ 0,\;x\leq 0 \end{array}\right.$$

The ReLU activation function outputs zero when its input is less than zero and equals its input otherwise. To reduce computational load, down sampling was performed in the pooling layer, where max pooling was used to extract the primary feature vector and enhance learning efficiency. A 2 × 2 filter was applied to select the maximum values, reducing the dimensions of the feature map by half. The second convolutional layer (Conv2) produces 32 feature maps using a 3 × 3 filter, with the ReLU activation function applied. To capture more complex features, the number of feature maps increased to 32. Max pooling with a 2 × 2 filter was again applied in the pooling layer. The third convolutional layer (Conv3) outputs 64 feature maps using a 3 × 3 filter, also utilizing the ReLU activation function. Max pooling with a 2 × 2 filter was used again in the pooling layer. In the fully connected layer, the 3D feature maps from the convolutional and pooling layers were flattened into a 1D vector. This layer consists of 512 nodes and employs the ReLU activation function. To prevent overfitting during training, a dropout rate of 0.5 was applied, randomly excluding some nodes [43]. The output layer contains a single node, using the sigmoid function for binary classification.

The sigmoid function is defined by the following equation [44]:

(2) $$y=\frac{1}{1+e^{-x}}$$

The model was optimized using the Adaptive Moment Estimation (ADAM) optimizer and trained with binary cross-entropy as the loss function. Training was conducted over 5 epochs with a batch size of 32.

Hyperparameter tuning was performed using the manual search method. Manual search, grid search, and random search are the three commonly used methods for hyperparameter selection [45]. Manual search involves researchers selecting hyperparameters based on their intuition and prior knowledge, allowing for a quick and straightforward approach to finding a reasonable solution [46].

### 2.4. Roasting Data Acquisition Device (RDAD)

The Roasting Data Acquisition Device (RDAD) is composed of both hardware and software components. The hardware includes an Arduino (Nano, Arduino Co., New York, NY, USA), a roaster, and several sensors configured as shown in Table 3.

A K-type thermocouple (RT-02K-M8, C-Linktech Co., Hoengseong-gun, Republic of Korea), mounted on a MAX6675 module (SZH-CH031, Analog Devices Inc., Wilmington, MA, USA), was used to measure temperature. K-type thermocouples offer a wide measurement range of 250–1100 °C. When combined with the MAX6675 module, which provides cold junction compensation, the measurement range is 0–1024 °C with a resolution of 0.25 °C. To monitor gas changes, the RDAD includes MQ-3 and MQ-7 sensors (NTREX Co., Ltd., Michuhol-gu, Incheon, Republic of Korea). The MQ-3 sensor is highly sensitive to alcohol gases and is characterized by its long lifespan and low cost [47], while the MQ-7 sensor is used to detect carbon monoxide (CO) and is known for its stability and longevity [48]. The RDAD was connected to a server via USB. The temperature and gas sensor values measured by the RDAD are monitored in real-time through communication with the six-axis robotic arm, enabling the robotic arm to control the roasting process. For this control, the camera mounted on the robotic arm is programmed to recognize the control buttons on the roaster through image processing and to directly manipulate these buttons by referencing stored images. Additionally, when device control is not required, the camera on the robotic arm continuously monitors the roasting status of the coffee beans. The RDAD setup is shown in Figure 2.

### 2.5. Food Process Robot Intelligent System (FPRIS)

FPRIS is an AI-based system designed to control the roaster by predicting the DoR in real time during the roasting process. Developed in Python, the FPRIS integrates the BFCNN model and the RDAD, both operating together on a workstation. A camera mounted on a six-axis robot arm moves to capture the roaster’s fryer. The six-axis arm is commanded to rotate. Every 0.2 s, an image is captured, the region of interest is cropped, and the image is saved to the file system. Images classified as non-coffee beans by the BFCNN are stored in a separate file system for further retraining. For images classified as coffee beans, the Sum of Pixel Grayscale Values (SPGV) is calculated, and the grayscale histogram is analyzed. The grayscale histogram of non-coffee images was also analyzed to compare differences between images.

The SPGV is defined by the following equation:

(3) $$\sum_{i=0}^{n-1}\sum_{j=0}^{n-1}G_{i,j}=G_{0,0}+\cdots +G_{i,j}+\cdots +G_{n-1,n-1}$$

n is the image size, i is the row index, j is the column index, and G is the grayscale value. The FPRIS collects gas data from the RDAD every 2 s. The collected data are then calibrated against the initial collected gas data.

The calibration was defined by the following equation:

(4) $$GID=v_{n}-v_{0}$$

**Figure 5:**
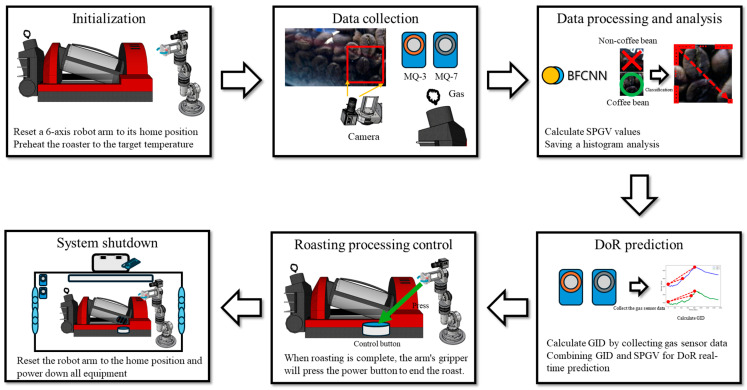
The flow of FPRIS.

GID means gas sensor initial difference, $v_{n}$ was the value of the gas sensor collected every 2 s, and $v_{0}$ was the initial value of the gas sensor. The initial difference between MQ-3 and MQ-7 was labeled as MQ3 GID, MQ7 GID. When the values being monitored reach the indicated DoR, the gripper, the end-effector of the six-axis robot arm, is commanded to press the control button on the roaster to end the roast. The overall flow of FPRIS is shown in Figure 5.

#### 2.5.1. Preliminary Procedures for FPRIS Operation

The roaster was preheated to 170 °C, and 100 g of green coffee were loaded. The Preliminary Coffee Sample (PCS) was then roasted according to the conditions shown in Table 4.

After roasting, it was cooled with forced air until it reached 60 °C. The weight loss due to roasting was measured and expressed as a percentage of the pre-roasting sample weight [49].

The weight loss (%) was calculated using the following formula:

(5) $$\begin{array}{l} Weight\;loss\;\left(\%\right)\\ =\frac{Initial\;weight\;of\;coffee\;bean-Final\;weight\;of\;coffee\;bean}{Initial\;weight\;of\;coffee\;bean}\times 100 \end{array}$$

#### 2.5.2. Procedures for FPRIS Validation

To validate the FPRIS system, the roaster was preheated to 170 °C, and 100 g of green coffee were loaded, as in the preliminary procedures. For this validation procedure, the roaster temperature was set to 215 °C to match the conditions required for validation. Unlike the preliminary procedure, the roasting process was monitored using FPRIS data, and the roasting was stopped when the DoR, as predicted by the FPRIS system, was reached. The Validation Coffee Sample (VCS) was then cooled with forced air until it reached 60 °C. The weight loss was measured and calculated to verify the accuracy of the FPRIS prediction.

### 2.6. Chromaticity Analysis of Coffee Samples

For both the PCS and the VCS, 20 g of green coffee beans and roasted samples were ground using a grinder (KG7070, Braun Co., Gimpo-si, Republic of Korea) with a one-step adjustment. The ground beans were placed in a glass shaker (DS101060K, Hwankyung-tech Co., Jongno-gu, Seoul, Republic of Korea) and pressed with a tamper. L* (lightness), a* (redness), and b* (yellowness) values were measured in triplicate using a colorimeter (TES-135A, Tes Electrical Electronic Corp., Taipei, Taiwan) and expressed as the average value. A calibration plate with standard color values (L* = 96.19, a* = 0.195, b* = 0.098) was used as the reference for measurements.

### 2.7. Statistical Analysis

A statistical analysis was performed using R-Studio (Version 4.3.1, Boston, MA, USA). The weight loss, MQ3 GID, MQ7 GID, and SPGV of the PCS and VCS were compared using Student’s *t*-test, and their significance was tested using one-way analysis of variance (ANOVA) and Duncan’s multiple range test. The colors of the PCS and the VCS were also tested for significance using one-way ANOVA and Duncan’s multiple range test.

## 3. Results and Discussion

### 3.1. The BFCNN Training Result

**Figure 6:**
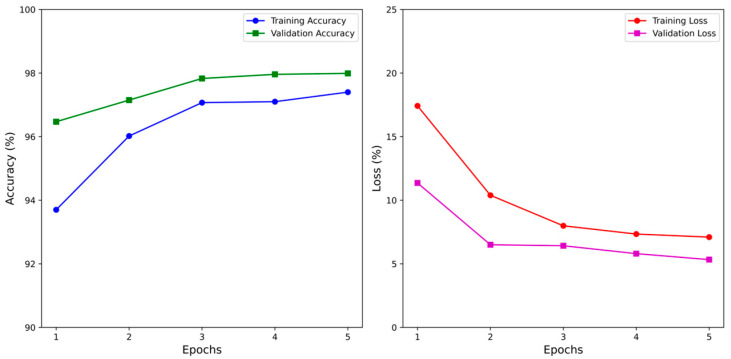
The training results of BFCNN.

The BFCNN was trained on a dataset with a batch size of 32 for 5 epochs, totaling 30 min and 33 s. The training dataset included 11,940 samples, and the validation dataset included 1492 samples. Figure 6 shows the training and validation accuracy and loss curves. The model achieved a maximum training accuracy of 97.40% and a minimum training loss of 7.10%. For validation, the maximum accuracy was 97.99%, with a minimum loss of 5.33%. Testing on a dataset of 1494 samples resulted in an accuracy of 97.59% and a loss of 5.58%.

### 3.2. Grayscale Histogram Comparison of Coffee and Non-Coffee Images

**Figure 7:**
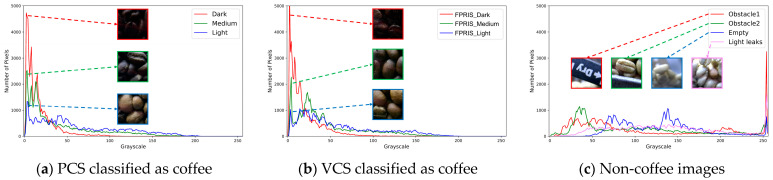
Grayscale histogram.

The grayscale histogram analysis of PCS and VCS coffee images (Figure 7a,b) shows a high density of pixels with grayscale values below 50. Additionally, as the roasting stage progresses from light to dark, the number of pixels near zero grayscale increases, resulting in a higher density in darker areas. This trend reflects the darkening color of coffee beans as roasting intensity increases. For non-coffee bean images, such as obstacles, empty spaces, and light leaks, histogram analysis (Figure 7c) shows a high concentration of pixels with grayscale values above 250. This pattern indicates that non-coffee images are characterized by higher grayscale values, suggesting that the BFCNN model can effectively distinguish between coffee and non-coffee images based on these characteristics.

### 3.3. Comparative Analysis of PCS and VCS Data

**Figure 8:**
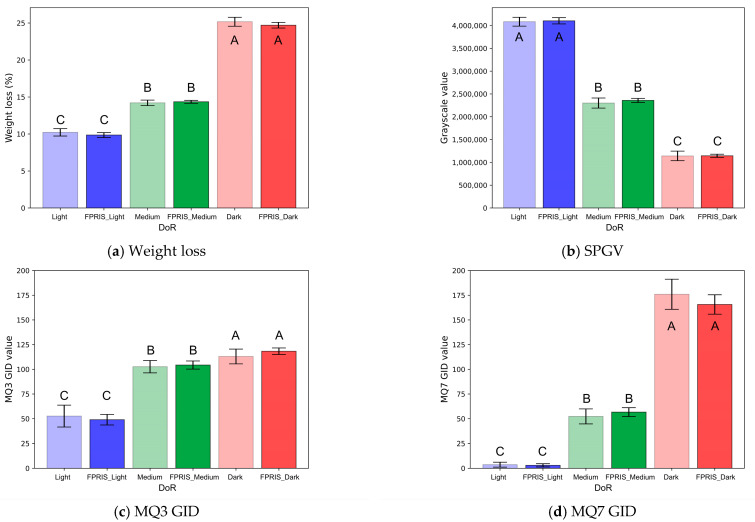
Comparative analysis results of PCS and VCS data. (The results of this study are expressed as the mean of triplicate samples with mean ± standard deviation. The error bars in the graph represent the standard deviation for each sample, visualizing the variability within repeated measurements. Values with different letters [A, B, C] within the same column are significantly different [*p* < 0.05] according to Duncan’s multiple range test. Statistical significance is indicated by *p* < 0.05, *p* < 0.01, and *p* < 0.001.)

A comparative analysis of PCS and VCS data is shown in Figure 8. In Figure 8a, both PCS (Light, Medium, Dark) and VCS (FPRIS_Light, FPRIS_Medium, FPRIS_Dark) are shown to have an increasing trend (*p* < 0.001) for weight loss as roasting intensifies. In a study by [50], the weight loss of coffee beans during roasting was attributed primarily to water evaporation in the early stages and to a series of chemical reactions (Maillard reaction, caramelization, lipid oxidation) in the later stages. It was also mentioned that the DoR of coffee beans can be classified based on weight loss. The weight loss was not significantly different between PCS and VCS (*p* ≥ 0.5). The SPGV analysis (Figure 8b) showed that increasing roasting intensity was associated with lower SPGV values for both PCS and VCS (*p* < 0.001). This reflects the darkening color of coffee beans, which becomes more pronounced as roasting progresses from Light to Dark DoR. This result suggests that FPRIS can be used to quantify DoR. Additionally, SPGV was not significantly different between PCS and VCS (*p* ≥ 0.5). The analysis of gas data (GID) revealed significant differences. Based on MQ-3 and MQ-7 sensor data (Figure 8c,d), GID values increased as roasting intensity increased (*p* < 0.001). GID values were significantly higher in the dark roasting stage. In [51], the investigators used MQ-3 gas sensors to measure the concentration of organic vapors emitted from coffee beans during roasting. The values from the MQ-3 gas sensor increased as the roast intensified, and the DoR was set based on this pattern, which is similar to the results of this study. Studies have shown that the heat generated during the roasting process causes certain compounds in the beans to evaporate, oxidize, or decompose, resulting in byproducts such as CO, NOx, and SOx. The MQ-7 gas sensor values detecting CO were highest at the end of roasting, aligning with a previous study [52]. Ref. [53] reported that CO emissions require ventilation monitoring and management in storage spaces to maintain CO concentrations at safe levels. From a safety perspective, FPRIS provides a means of monitoring CO concentrations. This trend is consistent for both PCS and VCS, suggesting that FPRIS effectively measures gas changes associated with roasting intensity.

### 3.4. Comparative Analysis of L*, a*, and b* Values Between PCS and VCS

**Figure 9:**
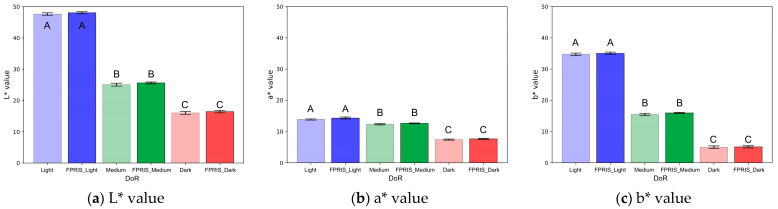
Chromaticity analysis results of PCS and VCS. (The results of this study are expressed as the mean of triplicate samples with mean ± standard deviation. The error bars in the graph represent the standard deviation for each sample, visualizing the variability within repeated measurements. Values with different letters [A, B, C] within the same column are significantly different [*p* < 0.05] according to Duncan’s multiple range test. Statistical significance is indicated by *p* < 0.05, *p* < 0.01, and *p* < 0.001).

The chromatic analysis results shown in Figure 9 indicate that L*, a*, and b* values tend to decrease significantly with increasing roasting intensity for both PCS and VCS. The L* values decreased significantly as roasting intensity increased from Light to Dark (*p* < 0.001), with no significant differences observed between PCS and VCS at any DoR level: Light (Light compared with FPRIS_Light), Medium (Medium compared with FPRIS_Medium), or Dark (Dark compared with FPRIS_Dark) (*p* ≥ 0.05) (Figure 9a). The a* values also decreased with increasing roasting intensity (*p* < 0.001), with no significant differences observed between PCS and VCS (*p* ≥ 0.05) (Figure 9b). The b* values significantly decreased with increasing roasting intensity (*p* < 0.001), with no significant differences observed between PCS and VCS at any roasting stage (*p* ≥ 0.05) (Figure 9c). The L* value measures the DoR related to roasting time and temperature and serves as an important indicator for assessing color changes during the roasting process [54,55]. Ref. [56] showed that L* values decreased in all samples as roasting progressed, with corresponding decreases in a* and b* values. These results are consistent with previous studies, demonstrating that chromaticity values can serve as indicators of DoR, effectively quantifying color changes with roasting intensity.

## 4. Conclusions

This study examined the potential application of FPRIS to automate and optimize coffee roasting as a food process. FPRIS is designed to integrate a 3D-printed six-axis robotic arm, gas sensors, and image sensor data to classify images of coffee beans inside the process system in real time and to effectively avoid voids and obstacles within the rotating roaster. By integrating the collected image and gas sensor data, the quality of coffee beans was quantified in real time, demonstrating precise control over the DoR. The comparative analysis of PCS and VCS data consistently showed that as roasting intensity increased, weight loss increased, SPGV decreased, and GID values increased. In particular, analysis of L*, a*, and b* chromaticity values showed a decrease in all values as roasting intensity increased, indicating a trend toward darker coffee beans. In conclusion, this study suggests that an FPRIS system combining AI, robotics, and computer vision can enhance the efficiency and precision of the food process. In future research, further experiments under various food processing conditions will be conducted to evaluate the universality of FPRIS, potentially leading to a smart automation system applicable across the food industry. 

## Figures and Tables

**Table 1:** Specification of motors.

Motor Model	Rated Voltage (V)	Rated Current (A)	Rated Torque (N∙m)	Rated Speed (RPM)
AK70-10 KV100	DC 24	7.2	8.3	148
AK60-6 V1.1 KV80	DC 24	3.8	3	233
28BYJ-48	DC 5	0.15–0.2	0.3–0.5	15

**Table 2:** The 3D printing parameters for 6-axis robotic arm components.

Part Name	Printing Time (hh:mm:ss)	Material Weight (g)	Material Length (m)
A_base1	02:29:44	161.18	54.04
A_base2	03:19:27	206.15	69.12
A_base3	02:03:28	106.23	35.62
B_motor1_link1	00:46:35	33.85	11.35
B_motor1_link2	00:54:04	52.13	17.48
C_motor2_bracket	02:48:27	95.57	32.04
C_motor2_link1	00:46:13	33.84	11.35
C_motor2_link2	05:05:58	301.79	101.19
D_motor3_base	00:29:10	23.60	7.91
D_motor3_bracket	01:03:33	41.73	13.99
D_motor3_link	00:00:22	8.96	3.00
F_motor4_bracket	02:16:15	67.59	22.66
F_motor4_link1	00:22:56	9.46	3.17
F_motor4_link2	01:45:45	83.71	28.07
G_motor5_base	00:28:55	23.59	7.91
G_motor5_bracket	01:03:28	41.71	13.98
G_motor5_link	00:22:07	8.96	3.00
H_motor6_bracket	02:19:47	80.05	26.84
H_motor6_link1	00:22:52	9.46	3.17
H_motor6_link2	01:06:49	41.71	13.98
I_gear	00:10:11	3.29	1.10
I_gripper	00:10:15	4.03	1.35
I_gripper_bracket	00:47:40	26.82	8.99
I_gripper_case	00:17:33	12.86	4.31
I_gripper_rack	00:12:56	6.28	2.11
I_stepper_base	00:14:24	9.40	3.15
I_stepper_case	00:55:51	28.57	9.58

**Table 3:** The 3D printing parameters for 6-axis robotic arm components.

Sensor	Measurement	Sensitivity
K-type thermocouple	Temperature	250–1100 °C
MAX6675	Temperature	0–1024 °C
MQ-3	Alcohol	25–500 ppm
MQ-7	Carbon monoxide	20–2000 ppm

**Table 4:** Temperature and time conditions for DoR.

DoR	Temperature (°C)	Time (min)
Light	200	9
Medium	215	13
Dark	225	21

## Data Availability

The original contributions presented in the study are included in the article; further inquiries can be directed to the corresponding author.
